# Within trial comparison of survival time projections from short‐term follow‐up with long‐term follow‐up findings

**DOI:** 10.1002/ehf2.13731

**Published:** 2022-07-07

**Authors:** João Pedro Ferreira, Brian L. Claggett, Kieran F. Docherty, Pardeep S. Jhund, Faiez Zannad, Scott D. Solomon, John J.V. McMurray

**Affiliations:** ^1^ INSERM, Centre d'Investigations Cliniques—1433, INI CRCT, INSERM U1116, CHRU Nancy Université de Lorraine Nancy France; ^2^ British Heart Foundation Cardiovascular Research Centre University of Glasgow 126 University Place Glasgow G12 8TA UK; ^3^ Cardiovascular Division Brigham and Women's Hospital, Harvard Medical School Boston MA USA

**Keywords:** Restricted mean survival time, Long‐term follow‐up, Survival estimates, Randomized controlled trials

## Abstract

**Aims:**

Data on long‐term treatment effects are scarce, despite the intent to use new therapies for many years and the need of patients, physicians and payers to have a better understanding of the lifetime benefits of treatments. The restricted mean or median survival time (RMST) calculated using age instead of time, hypothetically enables estimation of long‐term gain in event‐free or overall survival from the short‐term (within‐trial) effects of an intervention, compared with its control. Tha aim of the study is to use trials with long‐term follow‐up available through extension studies to compare the long‐term projections estimated using RMST from within‐trial follow‐up data with the actual long‐term outcomes in the extension studies.

**Methods and results:**

We estimated the median long‐term survival time using age instead of follow‐up time and compared these model‐based projections with the actual long‐term estimates in the (i) SCD‐HeFT trial vs. SCD‐HeFT long‐term outcomes; (ii) SOLVD trial vs. SOLVD 12 year follow‐up; (iii) STICH trial vs. STICHES; and (iv) ACCORD study vs. ACCORDION. In the long‐term follow‐up of SCD‐HeFT, gain in survival with ICD vs. placebo over a median of 11.0 years was +1.4 years of life. The RMST model‐derived survival projection from the within‐trial data (median follow‐up of 3.4 years) gave an estimated survival gain of +1.2 years. In STICHES, over a median follow‐up of 9.8 years, coronary artery bypass grafting (CABG) vs. medical care led to a survival extension of +1.4 years in favour of CABG. RMST projections using within‐trial data from STICH (median follow‐up of 4.9 years), gave an extended survival of +2.4 years in favour of CABG in younger patients. In the long‐term follow‐up of SOLVD, enalapril vs. placebo led to a survival gain of +0.8 years over a median follow‐up of 12.1 years. The RMST projections from the within‐trial data (median follow‐up of 2.8 years) gave a survival extension of +0.3 years in favour of enalapril. In the long‐term follow‐up ACCORDION study, with a median follow‐up of 8.8 years, intensive vs. a standard anti‐hyperglycaemic treatment did not influence long‐term survival, which was concordant with the RMST projections from the short‐term ACCORD study with median follow‐up of 4.9 years.

**Conclusions:**

Age‐based survival projections using within‐trial data generally provided concordant results with the actual survival measured in long‐term follow‐up extension studies. Our findings suggest that age‐based lifetime projections may be used as means to assess the long‐term treatment effects.

## Background

Randomized controlled trials are the best means of evaluating the efficacy and safety of treatments.^1^ However, most trials last for a relatively short period of time, often with a median follow‐up of only 1.5 to 3 years. This reflects the imperative to obtain results quickly, the cost of extending follow‐up and the difficulties in following patients and maintaining adherence to treatment over long periods of time. As a result, data on the long‐term treatment effects are scarce, despite the intent to use new therapies for many years and the desire of patients, physicians and payers require a better understanding of the lifetime benefits of these treatments.^2^ This may be especially important for younger people or individuals with less advanced disease where long‐term gains may be considerably larger than the short‐term benefits.^3^

Calculation of the restricted mean or median survival time (RMST) using age instead of time, enables estimation of long‐term event‐free survival that can be expressed as lifetime gained or lost from a specific intervention used in a trial, compared with its control.^4^ Although the RMST with age instead of time represents a potential advance on more conventional approaches, long‐term projections from short‐term follow‐up data are still model‐driven and lack support from real long‐term follow‐up. Notwithstanding, several long‐term extension studies, continuing after formal conclusion of randomized trials, provide an opportunity to compare the model‐based long‐term projections with the actual findings seen in the long‐term extension studies.

## Aims

The aim of the study is to compare model‐based long‐term projections from short‐term follow‐up data with the actual findings reported in the long‐term extension studies using trials (available to us) with both short‐term and long‐term follow‐up reports, specifically (i) SCD‐HeFT trial vs. SCD‐HeFT long‐term outcomes, comparing implantable cardioverter‐defibrillator (ICD) therapy to amiodarone or placebo; (ii) SOLVD trial vs. SOLVD 12year follow‐up, comparing enalapril to placebo; (iii) STICH trial vs. STICHES, comparing coronary‐artery bypass grafting (CABG) to medical therapy alone; and (iv) ACCORD study vs. ACCORDION, comparing intensive versus standard glycaemic control (*refer to supporting information Figure* *S1* *for details*).

## Methods

We computed the median survival time using age (instead of follow‐up time) in each short‐term follow‐up trial. These estimations are obtained by multiplying the annual conditional survival probabilities of patients included in the studies starting from a specific age, projecting the expected duration of event‐free survival up to a determined age horizon. For example, a given patient randomized to treatment A entering the trial at age 55 and censored from trial at age 58, was 3 years in the trial with Treatment A. Another patient randomized to Treatment B, also entering the trial at age 55 but dying at age 56, he/she was 1 year in the trial with Treatment B. Thus, the difference of life extension between Treatments A and B for these patients entering the trial at age 55 is +2 years in favour of Treatment A. Averaging the differences in life‐extension between Treatments A and B across all patients in the trial entering at a given age gives the RMST. We projected the estimates from the 25th, 50th, and 75th age percentile within each trial. This way we provide survival time projections across a wide age range, which is more representative of the patient‐population of the trial. The difference in the median age gained or lost between the active treatment and its control is given by the area under the Kaplan–Meier survival curve.^3^, ^5^ The key statistical assumption required to obtain valid long‐term survival projections based on short‐term follow‐up data is that a patient's risk for a given event depends on age, treatment effect, and follow‐up time.^3^, ^6^ All analyses were conducted using Stata (version 16; StataCorp. 2019).

## Results

As seen in the *Table* *1* (please refer to the table legend for details), the long‐term survival projections approximated the long‐term survival estimates reported in the actual extension studies from each of the trials examined.

**Table 1:** Comparison of the median survival time seen in the extended follow‐up of the studied trials with the long‐term median survival projections calculated from the short‐term follow‐up data

Trial	Median survival (in years) reported in the extended follow‐up cohorts^a^	Median survival projections (in years) computed from the short‐term follow‐up data starting in the 25th percentile of ageb	Median survival projections (in years) computed from the short‐term follow‐up data starting in the 50th percentile of age^b^	Median survival projections (in years) computed from the short‐term follow‐up data starting in the 75th percentile of age^b^
**SCD‐HeFT**	**SCD‐HeFT long‐term outcomes**	**SCD‐HeFT**	**SCD‐HeFT**	**SCD‐HeFT**
ICD vs. placebo	ICD: 9.5 (8.7–10.5)	ICD: 13.1 (11.2 to 15.2)	ICD: 8.7 (6.5 to 10.5)	ICD: 7.1 (4.9 to 8.5)
	PBO: 8.1 (7.3–8.9)	PBO: 10.3 (8.3 to 12.3)	PBO: 7.2 (6.0 to 9.4)	PBO: 6.7 (5.3 to 8.9)
	Diff: +1.4	Diff: +2.8 (0.4 to 5.2)	Diff: +1.2 (−0.9 to 3.2)	Diff: +0.5 (−1.7 to 2.7)
Amiodarone vs. placebo	AMD: 9.4 (8.2–10.2)	AMD: 11.0 (7.8 to 12.7)	AMD: 6.7 (5.6 to 9.4)	AMD: 5.4 (4.1 to 6.9)
	PBO: 8.1 (7.3–8.9)	PBO: 10.3 (8.3 to 12.3)	PBO: 7.2 (6.0 to 9.4)	PBO: 6.7 (5.3 to 8.9)
	Diff: +1.3	Diff: +0.7 (−2.3 to 3.7)	Diff: +0.5 (−1.9 to 2.9)	Diff: −1.3 (−3.3 to 0.8)
**STICH** ^c^	**STICHES long‐term outcomes**	**STICH**	**STICH**	**STICH**
CABG vs. medical care	CABG: 7.7^d^	CABG: 9.0 (7.4 to 10.8)	CABG: 6.8 (5.7 to 8.2)	CABG: 7.4 (5.9 to 9.9)
	MC: 6.3^d^	MC: 6.6 (4.4 to 9.4)	MC: 8.1 (6.1 to 9.7)	MC: 5.6 (4.7 to 6.6)
	Diff: +1.4	Diff: +2.4 (−0.7 to 5.5)	Diff: −1.3 (−3.4 to 0.8)	Diff: +1.8 (−0.1 to 3.7)
**SOLVD**	**SOLVD 12 year follow‐up**	**SOLVD**	**SOLVD**	**SOLVD**
Enalapril vs. placebo	ENL: 11.1 (5.6 to 14.3)	ENL: 10.5 (9.5 to 11.5)	ENL: 7.7 (6.8 to 8.8)	ENL: 6.8 (6.0 to 7.4)
	PBO: 10.3 (4.7 to 14.0)	PBO: 8.4 (7.7 to 9.5)	PBO: 7.4 (6.4 to 8.3)	PBO: 6.3 (5.4 to 7.3)
	Diff: +0.8	Diff: +2.1 (0.7 to 3.5)	Diff: +0.3 (−0.9 to 1.6)	Diff: +0.5 (−0.8 to 1.8)
**ACCORD**	**ACCORDION long‐term outcomes**	**ACCORD**	**ACCORD**	**ACCORD**
Intensive vs. standard	INT: 11.7 (11.6 to 11.8)	INT: 12.7 (12.6 to 12.9)	INT: 9.1 (9.0 to 9.2)	INT: 4.5 (4.4 to 4.6)
	STD: 11.7 (11.6 to 11.9)	STD: 12.8 (12.7 to 13.0)	STD: 9.2 (9.1 to 9.3)	STD: 4.6 (4.5 to 4.7)
	Diff: 0^e^	Diff: −0.1 (−0.3 to 0.1)	Diff: −0.1 (−0.2 to 0.4)	Diff: −0.1 (−0.2 to 0.0)

^a^ The extended follow‐up (median and percentile 25 to 75) for each trial was: 11.0 (10.0 to 12.2) years in SCD‐HeFT long‐term outcomes; 9.8 (9.1 to 11.0) years in STICHES; 12.1 (11.4 to 13.0) years in SOLVD 12 year follow‐up; and 8.8 years in ACCORDION (confidence interval not reported). The short‐term follow‐up time (median and percentile 25 to 75) in each trial was: 3.4 (2.5 to 4.4) years in SCD‐HeFT; 4.9 (4.1 to 6.0) years in STICH; 2.8 (2.0 to 3.7) years in SOLVD; and 4.9 (4.1 to 5.7) years in ACCORD.

^b^ To represent the treatment effect across the age range of the patients included in the studied trials, we calculated the median survival projections for any given patient starting the trial at the 25th, 50th (median), and 75th percentile of age within each trial; the median (percentile 25 to 75) age for each trial was: SCD‐HeFT: 60 (54 to 67) years; STICH: 60 (53 to 67) years; SOLVD: 60 (53 to 67) years; ACCORD: 62 (58 to 67) years. ACCORD has a ‘right‐skewed’ age distribution; thus, we have limited the projections up to the 90th percentile of age (72 years).

^c^ Consistent with the long‐term projections that we obtained from STICH short‐term follow‐up, in STICHES long‐term follow‐up study, a greater reduction in all‐cause mortality with CABG (vs. medical care) was seen in younger compared with older patients (interaction *P* = 0.062): Petrie et al.^7^

^d^ Confidence interval not provided.

^e^ No effect seen in long‐term survival (*P* = 0.91).

Legend: Diff., median survival time difference between the active and the control groups, that is, treatment—placebo/control; ICD, implantable cardioverter defibrillator; PBO, placebo; CABG, coronary artery bypass grafting; MC, medical care; AMD, amiodarone; ENL, enalapril; INT, intensive anti‐hyperglycaemic treatment; STD, standard anti‐hyperglycaemic treatment.

In SCD‐HeFT, the reported long‐term survival over a median follow‐up of 11.0 years was +1.4 years of life in favour of ICD (vs. placebo). The RMST model‐derived survival estimate from the short‐term follow‐up data (median follow‐up time of 3.4 years) starting at the median trial age of 60 years gave +1.2 years in favour of ICD, with a longer projected survival for younger patients (25th percentile of age = 54 years) of +2.8 years, and a shorter projected survival for older patients (75th percentile of age = 67 years) of +0.5 years. A similar pattern was found for the amiodarone (vs. placebo) arm, but with shorter observed and projected survival estimates than those seen with ICD. In the long‐term follow‐up STICHES study, CABG (vs. medical care) led to a survival extension of +1.4 years over a median follow‐up of 9.8 years. Using RMST projections from short‐term data in the STICH study with a median follow‐up time of 4.9 years, CABG (vs. medical care) was associated with an extended survival of +2.4 years for younger patients (25th percentile of age = 53), but not for patients older than the median of 60 years (where the projected survival time was −1.3 years). This finding is consistent with the actual long‐term findings in STICHES, where a greater reduction in all‐cause mortality with CABG (vs. medical care) was seen in younger but not in older patients (interaction *P* = 0.062).^7^ In the long‐term follow‐up of the SOLVD study over a median follow‐up of 12.1 years, enalapril (vs. placebo) led to a survival gain of +0.8 years. The RMST projections from the short‐term follow‐up SOLVD study (median follow‐up of 2.8 years), gave an increased survival of +0.3 years for patients starting the trial with the median age (=60 years), with longer survival projections for younger patients (25th percentile of age = 53 years) of +2.1 years. In the long‐term follow‐up ACCORDION study (median follow‐up of 8.8 years), and intensive anti‐hyperglycaemic treatment (vs. a standard one) did not influence long‐term survival, which was consistent with the RMST projections from the short‐term ACCORD study (median follow‐up of 4.9 years). *Table* *1*. The RMST projection figures are presented in *Figure* *S1*.

## Conclusions

The age‐based survival projections and the actual long‐term follow‐up survival estimates generally provided concordant results, despite several factors that could have limited their concordance. Specifically, the extension cohorts were a subset of all patients randomized and, in the device and surgery trials, the effects of the intervention continued after completion of the trial, whereas in the pharmacological treatment trials the randomized therapy could have been discontinued at the end of the trial. Alternatively, treatment cross‐overs might have occurred with the active therapy started in the placebo group after trial completion. Therefore, the long‐term follow‐up treatment effect estimates in the pharmacological studies may be more biased than those in the device and surgery trials. Nonetheless, our results suggest that age‐based lifetime projections may be used as means to assess the long‐term treatment effects. This information could be incorporated routinely in the reports of clinical trials thus providing important information about the expected long‐term effects of a given therapy or intervention for patients, doctors, and payers.

## Conflict of interest

The authors have nothing to disclose with regard to this study.

## Funding

Prof McMurray is supported by a British Heart Foundation Centre of Research Excellence Grant RE/18/6/34217.

## Supporting information


**Figure S1.** RMST representation for each trial.
