# Supplementary material for: Within trial comparison of survival time projections from short‐term follow‐up with long‐term follow‐up findings
Source: ESC Heart Fail. 2022 Jul 7;9(5):3655–8. doi: 10.1002/ehf2.13731 (PMC9715817; doi:10.1002/ehf2.13731)
Supplement: Supplementary file 1 — Figure S1. RMST representation for each trial. [file EHF2-9-3655-s001.docx]

**Supplemental Material**

Supplemental Figure 1. RMST representation for each trial


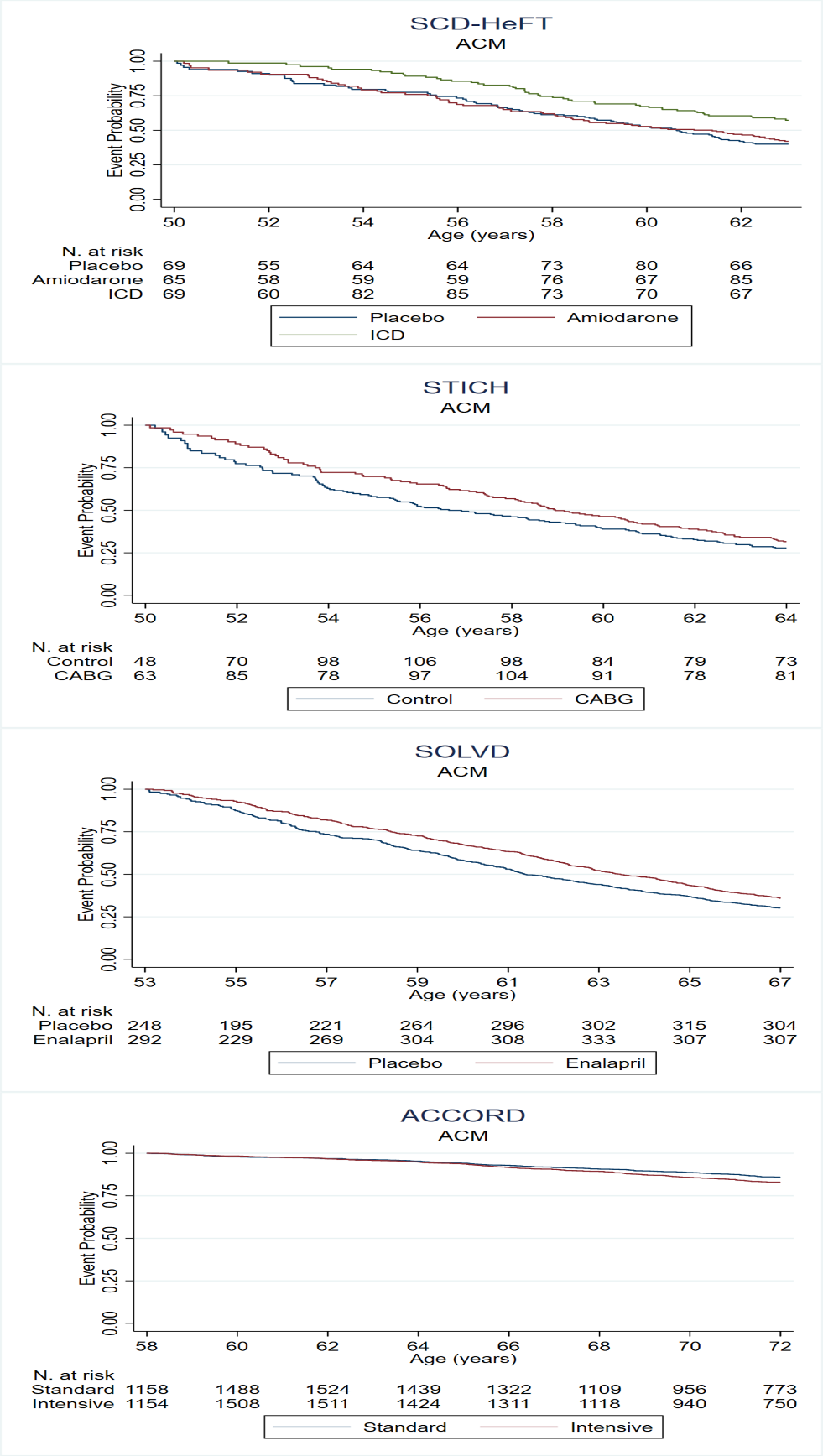


Legend: ACM, all-cause mortality.

The represented projections range from the 25^th^ to the 75^th^ percentile of age in each trial.

References to the trials used:

- The Sudden Cardiac Death in Heart Failure Trial (SCD-HeFT) trial:

Bardy, G. H.; Lee, K. L.; Mark, D. B.; Poole, J. E.; Packer, D. L.; Boineau, R.; Domanski, M.; Troutman, C.; Anderson, J.; Johnson, G.; McNulty, S. E.; Clapp-Channing, N.; Davidson-Ray, L. D.; Fraulo, E. S.; Fishbein, D. P.; Luceri, R. M.; Ip, J. H., Amiodarone or an implantable cardioverter-defibrillator for congestive heart failure. N Engl J Med 2005, 352 (3), 225-37.

Compared with

- The SCD-HeFT long-term outcomes:

Poole, J. E.; Olshansky, B.; Mark, D. B.; Anderson, J.; Johnson, G.; Hellkamp, A. S.; Davidson-Ray, L.; Fishbein, D. P.; Boineau, R. E.; Anstrom, K. J.; Reinhall, P. G.; Packer, D. L.; Lee, K. L.; Bardy, G. H., Long-Term Outcomes of Implantable Cardioverter-Defibrillator Therapy in the SCD-HeFT. J Am Coll Cardiol 2020, 76 (4), 405-415.

- The Studies of Left Ventricular Dysfunction (SOLVD) trial

Yusuf, S.; Pitt, B.; Davis, C. E.; Hood, W. B.; Cohn, J. N., Effect of enalapril on survival in patients with reduced left ventricular ejection fractions and congestive heart failure. N Engl J Med 1991, 325 (5), 293-302.

Compared with

- The SOLVD 12-year follow-up

Jong, P.; Yusuf, S.; Rousseau, M. F.; Ahn, S. A.; Bangdiwala, S. I., Effect of enalapril on 12-year survival and life expectancy in patients with left ventricular systolic dysfunction: a follow-up study. Lancet 2003, 361 (9372), 1843-8.

- The Surgical Treatment for Ischemic Heart Failure (STICH) trial

Velazquez, E. J.; Lee, K. L.; Deja, M. A.; Jain, A.; Sopko, G.; Marchenko, A.; Ali, I. S.; Pohost, G.; Gradinac, S.; Abraham, W. T.; Yii, M.; Prabhakaran, D.; Szwed, H.; Ferrazzi, P.; Petrie, M. C.; O'Connor, C. M.; Panchavinnin, P.; She, L.; Bonow, R. O.; Rankin, G. R.; Jones, R. H.; Rouleau, J. L., Coronary-artery bypass surgery in patients with left ventricular dysfunction. N Engl J Med 2011, 364 (17), 1607-16.

Compared with

- The STICH Extension Study (STICHES),

Velazquez, E. J.; Lee, K. L.; Jones, R. H.; Al-Khalidi, H. R.; Hill, J. A.; Panza, J. A.; Michler, R. E.; Bonow, R. O.; Doenst, T.; Petrie, M. C.; Oh, J. K.; She, L.; Moore, V. L.; Desvigne-Nickens, P.; Sopko, G.; Rouleau, J. L., Coronary-Artery Bypass Surgery in Patients with Ischemic Cardiomyopathy. N Engl J Med 2016, 374 (16), 1511-20.

- The Action to Control Cardiovascular Risk in Diabetes (ACCORD) study

Gerstein, H. C.; Miller, M. E.; Byington, R. P.; Goff, D. C., Jr.; Bigger, J. T.; Buse, J. B.; Cushman, W. C.; Genuth, S.; Ismail-Beigi, F.; Grimm, R. H., Jr.; Probstfield, J. L.; Simons-Morton, D. G.; Friedewald, W. T., Effects of intensive glucose lowering in type 2 diabetes. N Engl J Med 2008, 358 (24), 2545-59.

Compared with

- The ACCORD follow-on (ACCORDION) study

Nine-Year Effects of 3.7 Years of Intensive Glycemic Control on Cardiovascular Outcomes. Diabetes Care 2016, 39 (5), 701-8:12.
